# Association between suicidal risks and medication-overuse headache in chronic migraine: a cross-sectional study

**DOI:** 10.1186/s10194-021-01248-0

**Published:** 2021-05-10

**Authors:** Yen-Feng Wang, Chia-Chun Yu, Ai Seon Kuan, Shih-Pin Chen, Shuu-Jiun Wang

**Affiliations:** 1 Department of Neurology, Neurological Institute, Taipei Veterans General Hospital, Taipei, Taiwan; 2 School of Medicine, National Yang Ming Chiao Tung University College of Medicine, Taipei, Taiwan; 3 Brain Research Center, National Yang Ming Chiao Tung University, Taipei, Taiwan; 4 Department Neurosurgery, Neurological Institute, Taipei Veterans General Hospital, Taipei, Taiwan; 5 Institute of Public Health, National Yang Ming Chiao Tung University, Taipei, Taiwan; 6 Division of Translational Research, Department of Medical Research, Taipei Veterans General Hospital, Taipei, Taiwan

**Keywords:** Migraine, Substance dependence, Medication-overuse headache, Suicide

## Abstract

**Background:**

Behaviors of substance dependence are common among patients with medication-overuse headache (MOH). Whether MOH, like other substance use disorders, is associated with an increased risk for suicide is unknown.

**Methods:**

In this cross-sectional study, newly diagnosed chronic migraine (CM) patients with or without coexisting MOH were enrolled prospectively. Headache diagnoses were made through face-to-face interviews by headache specialists, and a specifically designed questionnaire was used to collect demographics, headache profiles, Migraine Disability Assessment, Hospital Anxiety and Depression Scale, Pittsburgh Sleep Quality Index, etc. Suicidal ideation and prior suicide attempt were specifically questioned.

**Results:**

In total, 603 CM patients (485F/118M, mean age 42.03 ± 12.18 years) were recruited, including 320 with MOH (257F/63M, mean age 42.8 ± 11.7 years) (53.1%), and 214 (35.5%) and 81 (13.4%) had suicidal ideation and prior suicide attempt, respectively. Among CM patients, the presence of MOH increased the risks of suicidal ideation (odds ratio [OR] = 1.75 [95% CI = 1.20–2.56], *p* = 0.004) and prior suicide attempt (OR = 1.88 [1.09–3.24], *p* = 0.024), after controlling for demographics, headache profile, disabilities, symptoms of anxiety and depression, and sleep quality.

**Conclusions:**

In CM patients, MOH is associated with an increased risk for suicidal ideation and prior suicide attempt, which deserves attention for clinicians taking care of headache patients. However, further studies are needed to determine the causal relationship, as well as the underlying pathophysiology.

## Background

Medication-overuse headache (MOH) is a secondary headache disorder resulting from long-term overuse of acute medications for migraine, and its prevalence was estimated at about 1% (range 0.5–7.2%) in the general population [1–8]. Prolonged excessive use of migraine acute medications could lead to worsening of the pre-existing primary headache disorders [7, 8]. In particular, overuse of acute medications is among one of the most important risk factors for migraine chronification [9–11]. MOH frequently coexists with chronic migraine (CM), and these two diagnoses are no longer mutually exclusive according to the Third Edition of the International Classification of Headache Disorders (ICHD-3) [12, 13]. MOH ranked among the most disabling neurological conditions according to the 2015 Global Burden of Disease study [14], and in addition to headache per se, MOH could be associated with psychiatric comorbidities [7], which makes the condition even more disabling.

Suicide is an important public health issue, and has been one of the leading causes of premature mortality, as estimated by years of life lost, around the world [15]. Patients with migraine and other chronic pain disorders are at increased risks for suicidal ideation or attempts [16–21]. Whether psychiatric comorbidities or certain psychological factors, such as mental turmoil, hopelessness, extreme sensory processing patterns, could be involved in the association remains to be elucidated [22–24]. On the other hand, suicide is also common in patients with substance use disorders involving alcohol, opioid, cocaine, etc., and up to 40% of patients who sought medical attention for substance use disorders had a history of suicide attempt [25]. It was also reported that cannabis use is associated with an increased risk for suicidal attempt and behavior [26], which could provide indirect evidence supportive of the association between substance use disorders and suicidal risks. The underlying mechanisms are uncertain, although reports on patients with alcohol use disorders suggested that comorbid depression is an important risk factor for suicide [27, 28]. Since two thirds of MOH patients could fulfill the criteria for substance dependence [29, 30], it could be hypothesized that MOH could share some clinical features with substance use disorders, including suicidal risks, and whether symptoms of depression or anxiety could have a role deserves exploration.

The primary objective of the present study is to compare the suicidal risks between CM patients with and without coexisting MOH, as well as to determine whether psychiatric comorbidities could be involved in the association between MOH and suicidal risks.

## Methods

### Patients

In this cross-sectional study, consecutive patients newly diagnosed with CM with or without a coexisting diagnosis of MOH were enrolled prospectively from the Headache Clinic of Taipei Veterans Hospital. Patients were recruited at their first visit, and were asked to complete a specifically designed questionnaire, which was followed by face-to-face interviews by headache specialists. The diagnoses of CM and MOH were made according to the ICHD-3 criteria [13]. Patients were included if they were (a) willing to participate in the study, (b) aged between 20 and 65 years, and (c) fulfilling the ICHD-3 criteria for CM. The exclusion criteria included (a) an acute headache disorder (within one month of headache onset), (b) a secondary headache disorder, and (c) difficulties completing history taking or questionnaire-based interview. The study protocol was approved by the Institutional Review Board of the Taipei Veterans General Hospital. All participants provided informed consent prior to participation.

### Questionnaire-based interview

The questionnaire was designed to collect the demographic and clinical characteristics of headache patients, and included general data, past medical and surgical histories, general physical condition, headache characteristics, medication use, psychological disturbances, etc. Headache-related disability was measured by using Migraine Disability Assessment (MIDAS), and moderate and severe disability was defined as MIDAS sore ≥ 11 [31]. MIDAS has been widely used in clinical studies and drug trials, and provides a measure for lost productivities related to headache attacks [32]. Symptoms of anxiety and depression were screened with Hospital Anxiety and Depression Scale (HADS), and depression and anxiety were defined as a depression score (HADS-D) ≥ 11 and an anxiety score (HADS-A) ≥ 11, respectively [33]. HADS is a 14-item instrument commonly used in the screening of anxiety and depressive disorders in the hospital setting [32, 33]. Sleep disturbances were assessed by using Pittsburgh Sleep Quality Index (PSQI), and a PSQI of > 5 was defined as poor sleep quality [34]. PSQI includes 19 self-rated questions designed to evaluate the quality and patterns of sleep in the previous month [34]. Lifetime suicidal ideation and attempt were assessed by two separate direct questions, i.e. “have you ever had ideational thoughts of engaging in suicidal behavior?” and have you ever had engaged in any self-injurious behavior with the intent to die?” These two questions, along with other parts of the questionnaire, were answered by the patients themselves, and the responses were confirmed at the time of subsequent face-to-face interviews by headache specialists.

### Statistical analysis

Descriptive data were expressed as mean ± standard deviation or number (percentages). Subjects with incomplete questionnaires, i.e. those with > 5% of the items unanswered, were excluded from analysis. Continuous variables, such as age, headache frequency, number of days with analgesic use, scores of neuropsychological instruments, etc. between groups were compared by using student’s t test. Chi-square test was used to compare categorical variables, such as sex, marital status, education level, employment status, suicidal ideation, prior suicide attempt, etc. Logistic regression modeling (backward stepwise, conditional) was carried out to estimate the odds ratios (ORs), as well the 95% confidence intervals (CIs), for suicide risks in patients with MOH as compared with those without, adjusted for potential confounders. All statistical analyses were carried out with SAS 9.4 (SAS Institute Inc., Cary, NC, USA). Statistical significance was defined as *p* < 0.05.

## Results

### Study participants

**Fig. 1:**
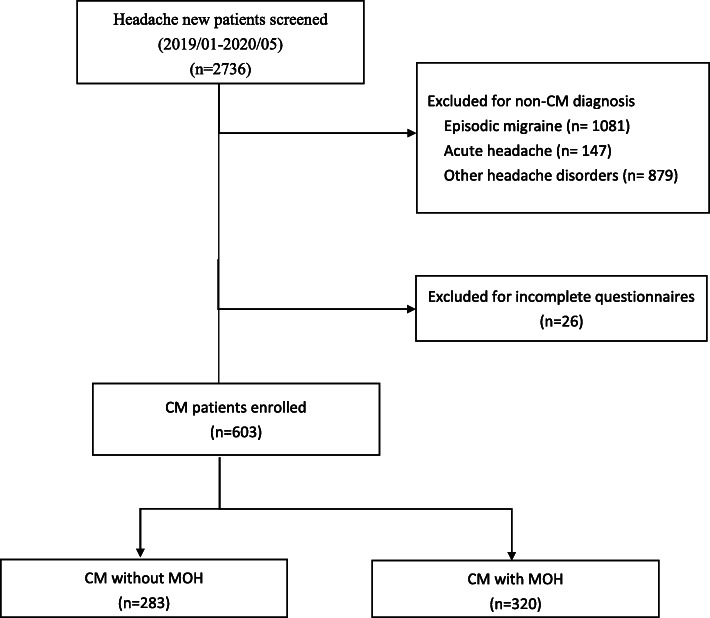
Patient recruitment in the present study

During the study period, 2736 consecutive headache patients (1924F/812M, mean age 41.41 ± 13.38 years, range 20–65) were screened at their first visit, and 2107 were excluded for diagnosis other than CM. Among patients diagnosed as CM, 26 were further excluded for incomplete questionnaires. In total, 603 CM patients (485F/118M, mean age 42.0 ± 12.2 years) were included for the analysis, consisting of 320 with MOH (257F/63M, mean age 42.8 ± 11.7 years) (53.1%) and 283 without (228F/55M, mean age 41.2 ± 12.7 years) (Table 1) (Fig. 1).

**Table 1:** Comparisons between chronic migraine patients with and without coexisting medication overuse headache (MOH)

	MOH (−) (*n* = 283)	MOH (+) (*n* = 320)	*p*-value
Age	41.2 ± 12.7	42.8 ± 11.7	0.093
Gender (female)	228 (80.6)	257 (80.3)	1
Education level			0.002
≤ High school	126 (44.5)	184 (57.5)	
≥ University	157 (55.5)	136 (42.5)	
Marital status			0.370
Unmarried^a^	146 (51.6)	153 (47.8)	
Married	137 (48.4)	167 (52.2)	
Employment status			0.580
Unemployed	98 (34.6)	104 (32.5)	
Employed	185 (65.4)	216 (67.5)	
Age at migraine onset	23.0 ± 10.9	20.7 ± 9.1	0.004
Aura	11 (3.9)	7 (2.2)	0.221
Headache frequency	22.4 ± 7.4	23.3 ± 6.9	0.098
Monthly analgesic use (days/month)	4.5 ± 6.3	19.4 ± 7.8	< 0.001
Moderate to severe disability (MIDAS ≥11)	198 (70.5)	240 (75.5)	0.168
Anxiety (HADS-A ≥ 11)	122 (43.1)	139 (43.7)	0.934
Depression (HADS-D ≥ 11)	71 (25.1)	93 (29.2)	0.272
Poor sleep (PSQI> 5)	254 (90.4)	285 (90.2)	1.000
Suicidal ideation	82 (29.4)	132 (41.6)	0.002
Suicide attempt	28 (10.1)	53 (16.7)	0.018

^a^Single, widowed, or divorced

*HADS* Hospital Anxiety and Depression Scale (A, anxiety subscale; D, depression subscale), *MIDAS* Migraine Disability Assessment, *MOH* medication-overuse headache, *PSQI* Pittsburgh Sleep Quality Index

### Comparisons between chronic migraine patients with and without medication overuse headache

Patients with MOH had lower education levels (high school or below: 57.5% vs. 44.5%, *p* = 0.002), earlier onset of migraine (mean age at onset: 20.7 ± 9.1 vs. 23.0 ± 10.9 years, *p* = 0.004), and more frequent analgesic use (19.4 ± 7.8 vs. 4.5 ± 6.3 days per month with analgesic use, *p* < 0.001), when compared with those without (Table 1). Besides, there was a trend toward an older age (mean age 42.8 ± 1.7 vs. 41.2 ± 12.7 years, *p* = 0.093), higher headache frequencies (23.3 ± 6.9 vs. 22.4 ± 7.4, *p* = 0.098) in MOH patients. However, the distributions of patient with moderate to severe disability (MIDAS ≥11) (75.5% vs. 70.5%, *p* = 0.168), depression (HADS-D ≥ 11) (29.2% vs. 25.1%, *p* = 0.272), anxiety (HADS-A ≥ 11) (43.7% vs. 43.1%, *p* = 0.934), or poor sleep quality (PSQI > 5) (90.2% vs. 90.4%, *p* = 1.000) were comparable.

### Medication-overuse headache as a risk factor for suicide

**Fig. 2:**
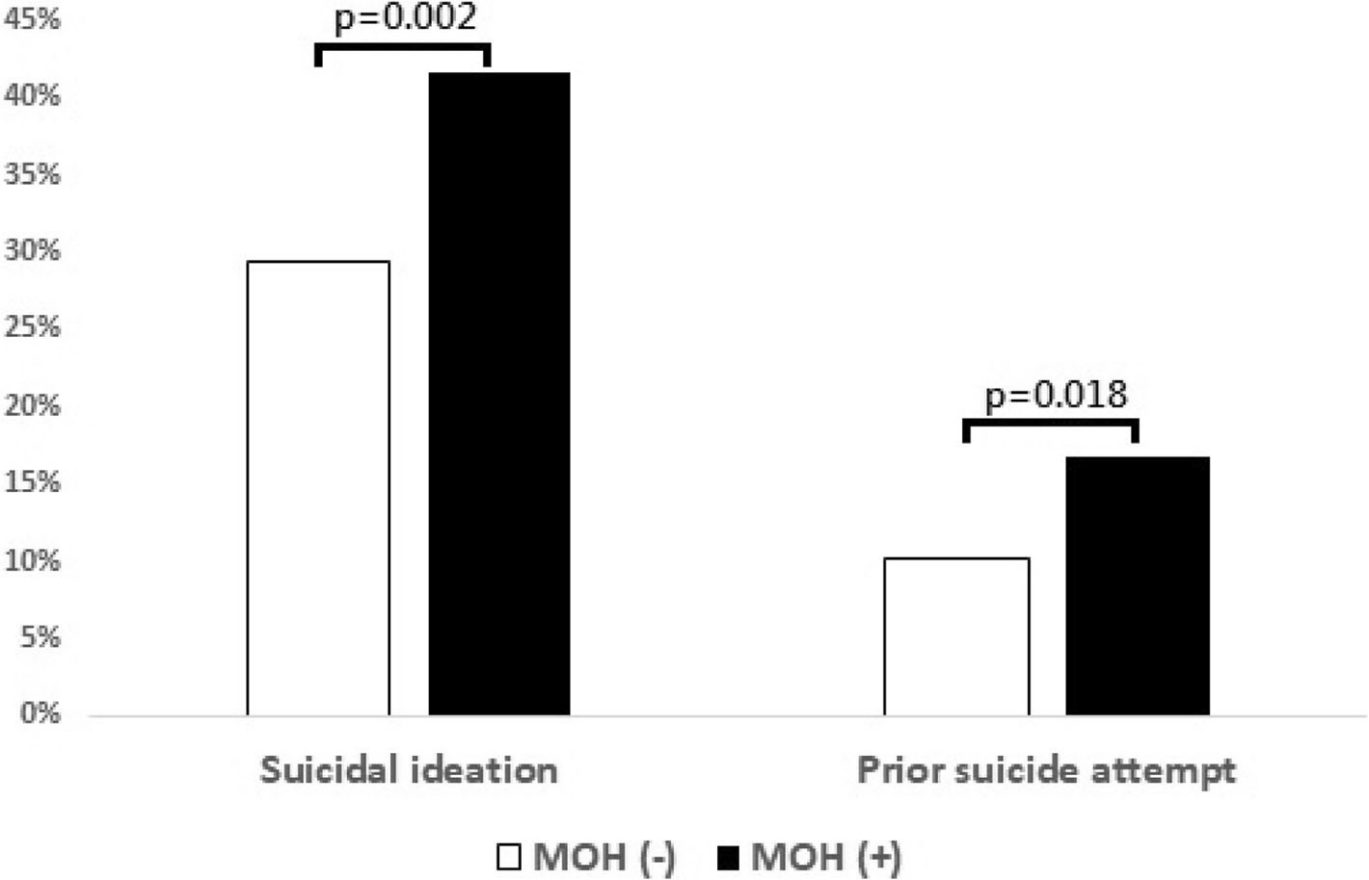
Proportions of patients with suicidal ideation and prior suicide attempt in chronic migraine patients with and without medication-overuse headache

Among the study participants, 214 (35.5%) and 81 (13.4%) had suicidal ideation and prior suicide attempt at some time in their lives, respectively. As compared with those without, CM patients with MOH were more likely to have suicidal ideation (41.6% vs. 29.4%, *p* = 0.002) and prior suicide attempt (16.7% vs. 10.1%, *p* = 0.018) (Fig. 2). Among CM patients, the presence of MOH was associated with increased risks of suicidal ideation (OR = 1.71 [95% CI = 1.22–2.41], p = 0.002) and prior suicide attempt (OR = 1.78 [1.10–2.92], *p* = 0.019). The associations remained significant after controlling for demographics, headache profile, migraine-related disability, and the presence of depression, anxiety, and poor sleep quality (suicidal ideation: OR = 1.75 [1.20–2.56], *p* = 0.004; prior suicide attempt: OR = 1.88 [1.09–3.24], *p* = 0.024) (Table 2).

**Table 2:** Comparative suicidal risks between chronic migraine (CM) patients with and without medication-overuse headache (MOH)

	Suicidal ideation		Prior suicide attempt	
	OR (95% CI)	*p*-value	OR (95% CI)	*p*-value
Crude	1.71 (1.22–2.41)	0.002	1.78 (1.10–2.92)	0.019
Model 1	1.77 (1.25–2.52)	0.001	1.75 (1.06–2.89)	0.028
Model 2	1.72 (1.20–2.46)	0.003	1.76 (1.06–2.94)	0.030
Model 3	1.75 (1.20–2.56)	0.004	1.88 (1.09–3.24)	0.024

Odds ratios between patients with and without MOH were estimated by using backward stepwise (conditional) logistic regression models. Demographics were controlled in Model 1, including age, gender, education level (≤ High school/≥ University), marital status (married/unmarried), employment status (unemployed/employed). In addition to demographics, headache profiles and disabilities were also controlled in Model 2, including age at migraine onset, aura status (yes/no), headache frequency, and the presence of at least moderate disability (MIDAS ≥11). Model 3 included demographics and headache profiles, as well as depression (HADS-D ≥ 11), anxiety (HADS-A ≥ 11), and poor sleep quality (PSQI> 5)

## Discussion

Among CM patients, patients with MOH were more likely to have suicidal ideation (41.6% vs. 29.4%, *p* = 0.002) and prior suicide attempt (16.7% vs. 10.1%, *p* = 0.018) (Fig. 2) when compared with those without. The presence of MOH was associated with increased risks for suicidal ideation (OR = 1.75 [95% CI = 1.20–2.56], *p* = 0.004) and suicide attempt (OR = 1.88 [1.09–3.24], *p* = 0.024), and the association was not accounted for by potential confounders, including demographics, headache profiles, migraine-related disability, psychiatric comorbidities, or sleep quality (Table 2). The findings highlight the importance of paying special attention to the risks of suicide in the management of patients with MOH.

One of the most important strengths is the relatively large sample size. More than 600 newly diagnosed CM patients with or without MOH were enrolled consecutively, which could potentially reduce selection bias. The data were of high quality and reliability. The diagnoses of CM and MOH were made through face-to-face interviews by experienced headache specialists, rather than just questionnaires, and well-validated and widely accepted neuropsychological instruments were utilized in the current study. Third, suicidal risks in MOH were assessed by using single direct questions followed by confirmation by the headache specialists, rather than psychiatric interviews. Such an approach could be more readily implemented in routine daily practice. Patients at risk could be efficiently identified and subsequently referred for formal psychiatric assessment. Therefore, the findings could have a more practical implication for neurologists.

In the current study, an association between MOH and suicidal risks was identified. In particular, comparisons were made between CM patients with and without MOH, and therefore, the finding were more likely to be pertinent to MOH per se. According to data of World Mental Health Surveys (WMHS), which were a series of national surveys for mental disorders by the World Health Organization, alcohol use disorders and drug use disorders were associated with increased risks for suicidal ideation (alcohol: OR = 2.0–2.5; drug: OR = 2.3–3.0) and attempt (alcohol: OR = 2.6–3.7; drug: OR = 2.0–4.0) [35, 36]. As MOH share many clinical features of substance use disorders [29, 30], the findings of the current study were actually in keeping with those in the WMHS, although suicidal risks in MOH were not as high as those in substance use disorders. Suicidal ideation and prior suicide attempts were reported to increase the risk of completed suicide [35, 37, 38], and identification of patients at risk is crucial in the prevention of suicide [39]. Besides, suicidal risks were found to be associated with compromised quality of life in patients with chronic daily headache (CDH) and MOH [22]. Therefore, it is important to be alert to the potential risk of suicide in taking care of patients with MOH.

The mechanisms underlying the association between MOH and suicide risks are unknown. It could be possible that psychiatric comorbidities might play a role. In particular, depressive and anxiety disorders are not uncommon in patients with migraine and CDH with MOH, and it could be possible that these psychiatric comorbidities could potentially participate in the association [22, 23]. However, the association between suicidal risks and MOH remained significant after controlling for depression and anxiety in the present cohort (Table 2). On the other hand, certain psychosocial factors shared by suicide, chronic pain, and even substance use disorders, could be involved, such as perceived burdensomeness, thwarted belongingness, defeat, etc. [40–44]. Besides, extreme sensory processing patterns implicated in emotional processes, depression, and suicidality could also have a role [24]. However, further studies are needed to clarify whether there could be mediating effects from these factors. Besides, since it is not uncommon for MOH patients to have concomitant overuse of tobacco, caffeine, or sedatives/anxiolytics [30], whether these centrally acting agents could also have a role remains to be determined. Further studies are needed to confirm our findings, as well as to explore the underlying mechanisms.

There are also some limitations. The study recruited patients from a tertiary referral center, and therefore, there could be concerns about the generalizability of the results. However, a relatively large cohort of consecutive patients were enrolled, which could potentially minimize selection bias. A formal referral system is being developed in the healthcare system of our country, and most of our patients accessed us directly without referral. Therefore, the results could reflect what could be seen in the general population to a certain extent. Second, the risk of suicidal ideation or prior suicide attempt was evaluated by two self-administered single-item direct questions rather than formal face-to-face interviews by psychiatrists, and the validity of such an approach is not without doubt [45, 46]. However, self-report was, in fact, demonstrated to be comparable to clinical interviews in suicide assessment [47]. Besides, even though single-item questions could lead to misclassification, the majority of patients with a positive response were still qualified as having increased suicidal risks [47]. Nevertheless, the lack of a validated measure for suicide remains an important concern. The findings were, in fact, extrapolated from the answers to these two self-administered questions at the time of clinical evaluation, and whether those with a positive response would really have suicidal ideation or behavior in the future needs to be further confirmed. Third, the levels of depression or anxiety, as defined by scores of HADS, rather than formal psychiatric diagnoses, were used in this study, which could potentially introduce some concerns. However, this approach has been commonly used and widely accepted in clinical studies involving questionnaires, and the cut-off scores were actually validated in clinical studies [33]. Fourth, patients with > 5% of the items in the questionnaire unanswered were excluded from the analysis, which could introduce some selection bias as such patients might have clinical profiles different from those included in the analysis. However, only a small proportion of patients were excluded for this reason (*n* = 26) (Fig. 1), which could have little impact on the results in the current study. Fifth, what the current study reported was an association between MOH and suicidality. The causal relationship could be further investigated by longitudinal studies involving MOH patients without prior suicidal ideation or attempt at baseline, and new-onset suicidal ideation or attempt at follow-up would help determine the causality or directionality of the association. Besides, complete psychological evaluations in these patients would help clarify the psychological mechanisms that could be involved.

## Conclusions

The present study identified an association between MOH and suicidal risks in patients with CM, suggestive of a potential link between behaviors of dependence and suicide. The potential risks of suicide should not be overlooked in clinical practice for clinicians taking care of patients with MOH. However, the causal relationship and underlying mechanisms are uncertain, and further longitudinal studies are needed to clarify the causal relationship and the directionality of the association.

## Data Availability

The datasets used and/or analyzed during the current study are available from the corresponding author on reasonable request.

## Abbreviations

CDH: Chronic daily headache
CI: Confidence interval
CM: Chronic migraine
HADS: Hospital Anxiety and Depression Scale
HADS-A: Anxiety subscale of Hospital Anxiety and Depression Scale
HADS-D: Depression subscale of Hospital Anxiety and Depression Scale
ICHD-3: Third Edition of the International Classification of Headache Disorders
MIDAS: Migraine Disability Assessment Scale
MOH: Medication-overuse headache
OR: Odds ratio
PSQI: Pittsburgh Sleep Quality Index
WMHS: World Mental Health Surveys
